# Predicting potential suitable habitat of *Cistanche deserticola* by integrating parasitic constraints and land use data into MaxEnt modeling

**DOI:** 10.3389/fpls.2025.1635595

**Published:** 2025-08-04

**Authors:** Gong-Han Tu, Xu-Dong Guo, Shao-Yang Xi, Xiao-Hui Ma, Ling Jin

**Affiliations:** College of Pharmacy, Gansu University of Chinese Medicine, Lanzhou, China

**Keywords:** *Cistanche deserticola*, MaxEnt, climate change, parasitic constraint scenario, land use

## Abstract

**Introduction:**

Understanding the impacts of climate change and land use dynamics on parasitic plants is crucial for ecological restoration and sustainable resource management in arid regions. This study proposes a two-dimensional modeling framework that integrates parasitic constraints and land use dynamics to predict the potential suitable habitat of *Cistanche deserticola*, a medicinal plant obligately parasitic on Haloxylon ammodendron.

**Methods:**

Using an optimized MaxEnt model, host suitability probability was incorporated as a continuous probabilistic constraint, and high-resolution land use data were coupled to enhance ecological realism. The framework was applied to assess habitat suitability under current (1970-2000) and future climate scenarios (2050s, 2070s, 2090s, SSP126, SSP370, SSP585).

**Results:**

The inclusion of parasitic constraints reduced the suitable habitat area by 4.5% (from 138.20 × 104 km² to 131.92 × 104 km²) and exacerbated habitat fragmentation, particularly in Northwest China. Future projections reveal a decrease in the total suitable habitat area but an increase in the area of highly suitable regions, with the centroid shifting towards the northwest. Land use analysis demonstrated that unused land (70.21%) and grassland (13.81%) constitute the primary habitats, highlighting their significance for sustainable cultivation. Key environmental drivers identified include July precipitation, soil pH, and temperature of the warmest quarter. The model exhibited high predictive accuracy (AUC: 0.947-0.949).

**Discussion:**

The framework provides a reliable tool for assessing host-parasite interactions and land use impacts. These findings offer valuable insights for adaptive management strategies that balance ecological restoration and the sustainability of medicinal resources in arid ecosystems.

## 1 Introduction

Climate change represents one of the most formidable environmental challenges globally, profoundly impacting arid ecosystems by altering precipitation patterns, increasing temperatures, and intensifying drought events. These changes, along with shifts in extreme weather events, seasonality, humidity, and evaporation rates, not only directly drive desertification processes but also significantly influence the structure and function of ecological networks (Huang and Zhai, 2023). According to the World Meteorological Organization (WMO), the frequency and intensity of global droughts have increased by approximately 29% over the past 50 years, leading to continuous expansion of land degradation and desertification, particularly in arid regions of Asia and Africa (Li et al., 2021). The resulting contraction of suitable habitats, shifts in species distribution ranges, and degradation of ecosystem functions are particularly pronounced in the arid regions of Northwest China, severely limiting the survival and reproduction of native species and threatening biodiversity and ecosystem stability (Huang and Zhai, 2023). Furthermore, the interaction between climate change and anthropogenic activities, such as nutrient fluctuations and land use change, further complicates ecological network dynamics. This reorganization of ecological networks reveals the profound impact of environmental stress on species interactions, offering theoretical insights for understanding the dynamic responses of critical ecological relationships, such as the obligate parasitism foundational to keystone species in arid regions (Merz et al., 2023; Wang et al., 2024).

Arid ecosystems, due to their inherent fragility, are particularly sensitive to environmental changes. It is important to note that this sensitivity can vary spatially along aridity gradients (e.g., semi-arid, arid, hyper-arid) depending on baseline conditions and ecosystem type. Within these systems, species interaction networks play a critical role in maintaining stability. A prime example is the unique and vital obligate holoparasitic relationship between *Cistanche deserticola* Y.C.Ma (*C. deserticola*), known as ‘Desert Ginseng’, and its exclusive host *Haloxylon ammodendron* (C. A. Mey.) Bunge (*H. ammodendron*). *H. ammodendron* is a key sand-fixing shrub widely distributed in arid regions of Central Asia, including Northwest China (Fan et al., 2023). This Cistanche-Haloxylon system is not only crucial for desertification control but also holds significant cultural and economic value due to *C. deserticola’s* esteemed status as a traditional Chinese medicine (Cheng et al., 2023; Zhang et al., 2024). However, the obligate nature of this specific host-parasite interaction, coupled with decades of unsustainable wild harvesting and intensifying climate change impacts, places immense pressure on *C. deserticola* populations. This extreme dependency of the parasite on its host means that the survival and distribution of *C. deserticola* are inherently limited by the ecological niche and spatial distribution of *H. ammodendron*. Despite its critical ecological and economic importance, existing ecological models often overlook the dynamic dependency between *C. deserticola* and its host, as well as the synergistic effects of land use change on this specific system. This oversight leads to systemic biases in the formulation of conservation strategies, limiting the comprehensive benefits of ecological restoration and sustainable resource utilization for this vulnerable species (Ma et al., 2023; Sarabeev et al., 2022).

Species distribution models (SDMs), particularly the Maximum Entropy model (MaxEnt), have been widely employed for the conservation of endangered species, assessment of climate change impacts on species distribution, ecological restoration, and the study of physiological processes underlying species-environment interactions, owing to their advantages in handling sparse distribution data and simulating complex ecological relationships (Xie et al., 2024; Lin et al., 2024; Hu et al., 2020; Gong et al., 2023). In recent years, the ecological realism of MaxEnt has been significantly enhanced through the integration of biological and abiotic factors, optimization of model parameters, and the incorporation of spatial constraints (Shao et al., 2022). For obligate parasitic species like *C. deserticola*, research has gradually shifted from models driven by single environmental factors towards complex models coupling biological interactions and spatial dynamics (Lu et al., 2022). Nevertheless, existing studies on this specific Cistanche-Haloxylon system, or similar obligate parasitic relationships, still exhibit limitations: firstly, the coupling mechanism between hosts and environmental factors lacks dynamic quantitative constraints, resulting in insufficient ecological representation of this strict host-parasite dependency in models; secondly, the impact of land use change on habitat suitability is often neglected, restricting the applicability of models in practical conservation and resource management, a critical oversight given the dual pressures of desertification and human activity on *C. deserticola’s* limited habitats.

Beyond its ecological dependency and medicinal value, the Cistanche-Haloxylon system also represents a unique ecological partnership; while *H. ammodendron* is vital for desertification control, *C. deserticola* parasitism has been shown to enhance the host’s drought tolerance and improve local soil conditions, contributing to the resilience of arid ecosystems (Fan et al., 2023; Miao et al., 2023b). The intertwined ecological and economic significance of this system, coupled with the strict host-parasite relationship and vulnerability to environmental change and human activity, underscores the urgent need for sophisticated modeling approaches to inform its conservation and sustainable management.

**Figure 1:**
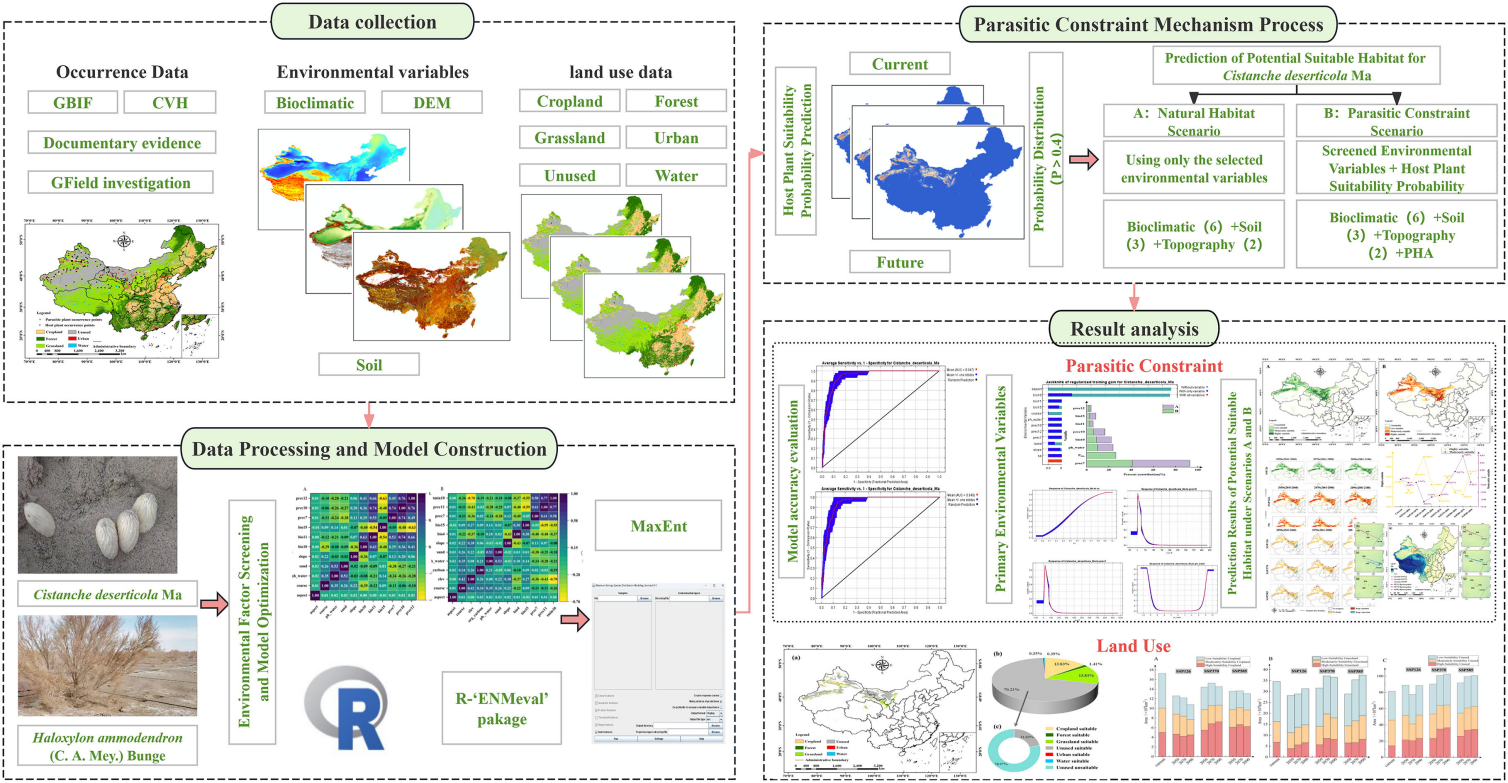
Schematic diagram of the study.

To address the critical limitations in current ecological modeling approaches for species with complex biological dependencies and to provide a more accurate and practical assessment for the conservation and sustainable utilization of *C. deserticola*, this study develops and applies a novel two-dimensional modeling framework. This framework moves beyond traditional methods by explicitly integrating a quantitative representation of host suitability as a continuous probabilistic constraint and incorporating high-resolution land use data to refine potential habitat predictions. Utilizing current climate data and multi-scenario future climate projections from CMIP6 (Shared Socioeconomic Pathways - SSPs), the spatiotemporal dynamics of potential suitable habitat of *C. deserticola* are systematically assessed. Specifically, this research aims to: develop and implement a method for incorporating the continuous probability gradient of host suitability as a dynamic constraint within the MaxEnt modeling process, thereby enhancing the ecological realism of predictions for obligate parasitic species; quantify and compare the predicted suitable habitat distributions and characteristics of *C. deserticola* under scenarios considering only environmental suitability (natural habitat scenario) versus those incorporating the host suitability constraint (parasitic constraint scenario); refine the predicted potential suitable areas by integrating current land use data to identify available and suitable habitats, providing a more practical basis for management; and finally, project the future distribution of suitable habitat under various climate change scenarios (SSP126, SSP370, SSP585) and analyze the relative influence of climate, host suitability, and land use on habitat suitability and shifts. The schematic diagram of the study is shown in **Figure 1**. The results are intended to provide critical, spatially explicit insights for developing adaptive management strategies that effectively balance ecological restoration, biodiversity conservation, and the sustainable utilization of medicinal plant resources in arid ecosystems, and offer a transferable modeling approach applicable to other species with complex biological dependencies in dynamic landscapes.

## 2 Materials and methods

### 2.1 Species occurrence data acquisition and processing

**Figure 2:**
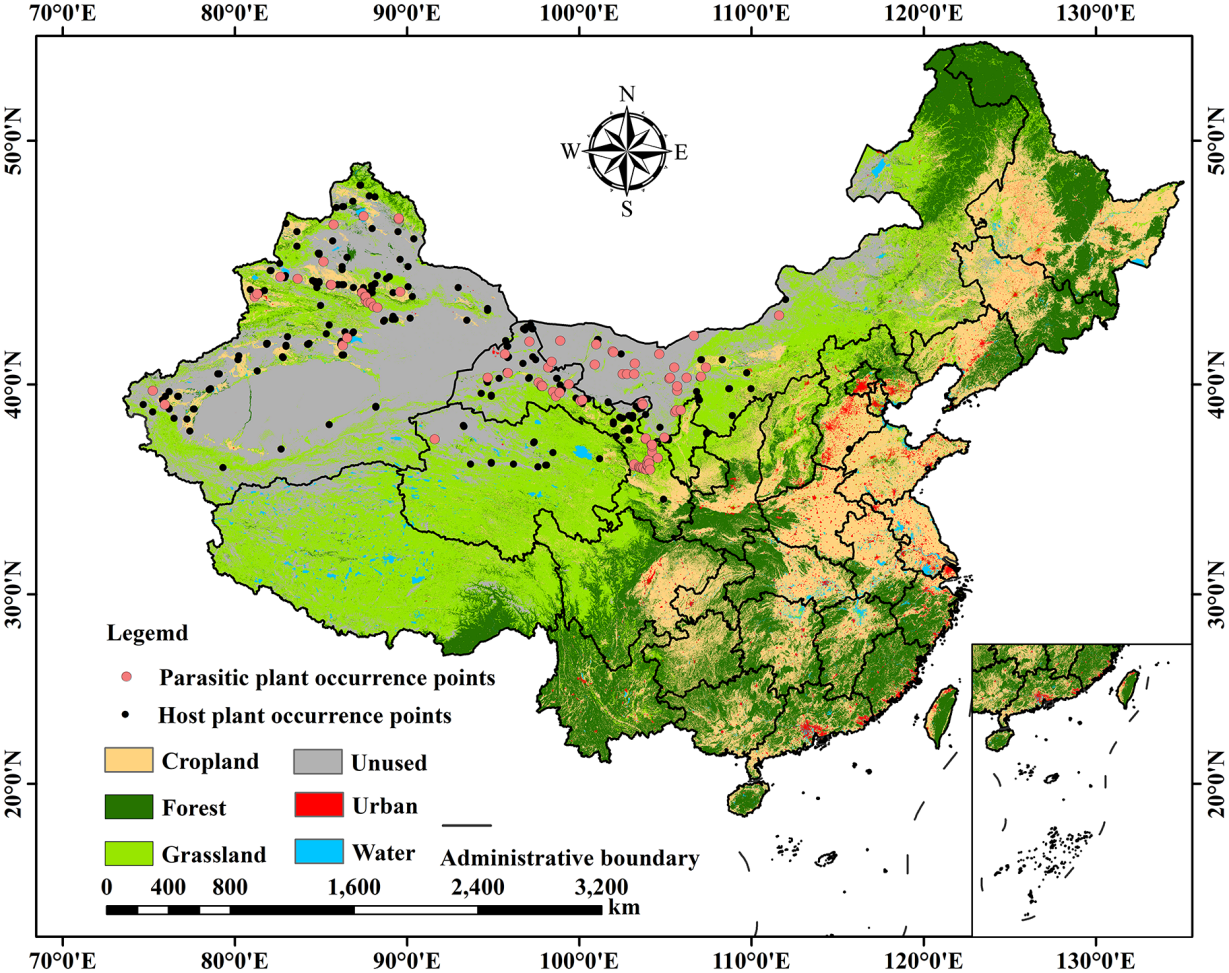
Occurrence points of the parasitic plant *C. deserticola* and its host plant *H. ammodendron*.

Geographic occurrence data for *C. deserticola* and its host plant *H. ammodendron* were compiled from multiple sources: the Global Biodiversity Information Facility (GBIF, https://www.gbif.org/zh/), the Chinese Virtual Herbarium (CVH, http://www.cvh.ac.cn/), dedicated field surveys, and relevant published literature. To mitigate potential spatial biases from opportunistic sampling and reduce the influence of spatially redundant data on predictive outcomes, the collected occurrence records underwent a rigorous filtering process. Initial removal of duplicate entries and apparent outliers was followed by spatial thinning using ENM Tools software (http://enmtools.blogspot.com/). This process, guided by the resolution of the environmental data, ensured only a single occurrence point remained within each 5 km×5 km grid cell. Additionally, any occurrence points located outside the territorial boundaries of China were excluded. Following this comprehensive filtering procedure, a final dataset comprising 88 occurrence points for C. deserticola and 234 points for H. ammodendron was obtained (**Figure 2**). These refined datasets were saved in.csv format for subsequent MaxEnt model construction (Xi et al., 2025). The base map of China utilized in this study was sourced from the Standard Map Service System of the Ministry of Natural Resources of China (http://bzdt.ch.mnr.gov.cn/), with the official approval number GS (2024) 0650.

### 2.2 Environmental variable selection and data processing

Current climate data for the period 1970–2000 and future climate data for the periods 2041–2060 (2050s), 2061–2080 (2070s), and 2081–2100 (2090s) were obtained from WorldClim (http://www.worldclim.org). This dataset comprised 19 bioclimatic variables (Bio1–19), 24 temperature variables (Tmax1–12, Tmin1–12), 12 precipitation variables (Prec1–12), and one topographic variable (elevation). All variables had a spatial resolution of 2.5 arc-minutes.

Future climate projections were based on the medium-resolution climate system model BCC-CSM2-MR from the China (Beijing) Climate Center, participating in the Coupled Model Intercomparison Project Phase 6 (CMIP6). Future climate change scenarios included three Shared Socioeconomic Pathways (SSPs): SSP126, SSP370, and SSP585 (Zhu et al., 2024). These scenarios represent distinct future trajectories: SSP126 represents a pathway with low vulnerability, low mitigation challenges, and low radiative forcing; SSP370 combines high social vulnerability with relatively high anthropogenic radiative forcing; and SSP585 depicts a high forcing scenario.

Slope and aspect data were derived from topographic data using ArcGIS 10.8. Additionally, five soil variables were obtained from the World Soil Database (v2.0, https://www.fao.org/soils-portal/data-hub/en/). For future scenario projections, it was assumed that topography and soil conditions would not undergo significant changes compared to the present (Xu et al., 2024).

**Figure 3:**
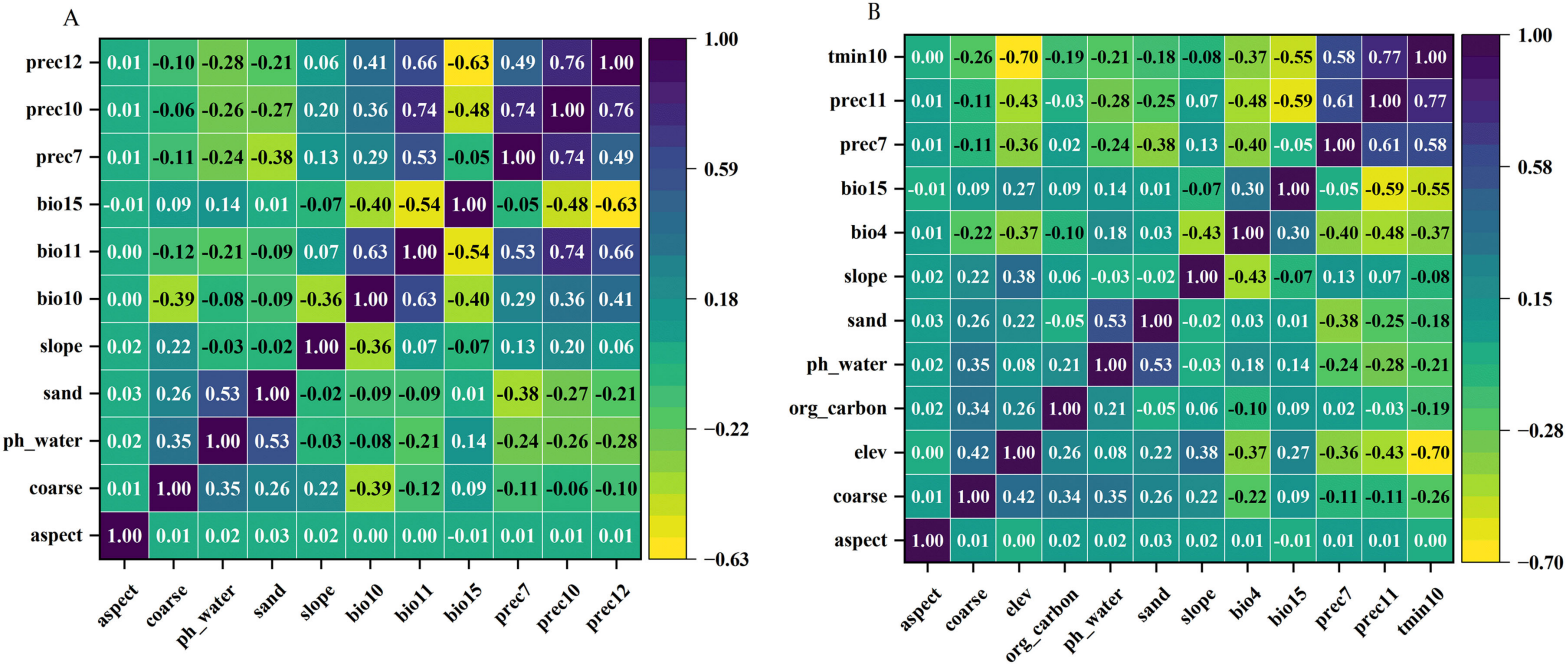
Correlation heatmap of filtered environmental variables for MaxEnt modeling. **(A)** Variables for *C*. *deserticola*. **(B)** Variables for *H*. *ammodendron*.

To prevent model overfitting caused by multicollinearity among environmental variables, a variable selection process was implemented. This process was based on the contribution of variables in preliminary MaxEnt runs and their correlation (|r|) calculated using ENM Tools for variables exhibiting a contribution greater than zero. Variables with a high correlation (|r| ≥ 0.8) and relatively low contribution were excluded (Zhao et al., 2024). The final set of selected environmental variables is presented in **Supplementary Table S1**, and their correlation matrix is shown in **Figure 3**.

### 2.3 Model optimization and evaluation

To address MaxEnt model susceptibility to overfitting, model tuning was performed using the ENMeval package (R version 4.3.3) (Muscarella et al., 2014). A range of Regularization Multipliers (RMs) from 0.5 to 4 (increments of 0.5, 8 values total) were tested with six Feature Combinations (FCs): L (linear), LQ (linear, quadratic), LQH (linear, quadratic, hinge), H (hinge), LQHP (linear, quadratic, hinge, product), and LQHPT (linear, quadratic, hinge, product, threshold). This resulted in a total of 48 parameter combinations evaluated within the ENMeval framework. The optimal parameter combination for subsequent MaxEnt modeling was identified as the one yielding a delta.AICc value of 0 (Wu et al., 2024) (**Table 1**). Using the optimized parameters, the MaxEnt model (version 3.4.1) was employed to predict potential suitable distribution areas for C. deserticola and H. ammodendron. Current occurrence data (.csv) and environmental data (.asc) were imported. The jackknife method was selected for model evaluation, and the output format was set to Logistic. Occurrence data were partitioned, with 25% for testing and 75% for training. The model was run 10 times with the selected optimized RM and FC values, while other parameters were kept at their default settings. Model accuracy was primarily assessed using the Area Under the Receiver Operating Characteristic curve (AUC) generated from the MaxEnt output (Wen et al., 2024). AUC values, ranging from 0 to 1, were interpreted based on standard thresholds: invalid (0.5 ≤ AUC < 0.6), poor (0.6 ≤ AUC < 0.7), average (0.7 ≤ AUC < 0.8), good (0.8 ≤ AUC < 0.9), and excellent (0.9 ≤ AUC ≤ 1) (Gao et al., 2024).

**Table 1:** Optimizing MaxEnt parameter settings using the ENMeval package.

Type	Species	FC	RM	delta.AICc
Default	*C. deserticola* Ma	LQPH	1	185.71
	*H. ammodendron* (C. A. Mey.) Bunge	LQPH	1	151.17
Optimized	*C. deserticola* Ma	LQHPT	3.5	0
	*H. ammodendron* (C. A. Mey.) Bunge	LQHPT	2.5	0

### 2.4 Suitable habitat classification and area calculation

The average output raster from the MaxEnt model was imported into ArcGIS. Using conversion tools, the data was transformed from.asc format to raster format. Based on the suitability index (*P*), the potential distribution area was classified into four suitability levels using a manual classification method. These levels were defined as follows: Unsuitable (*P* < 0.2), Low suitability (0.2 ≤ *P* < 0.4), Medium suitability (0.4 ≤ *P* < 0.6), and High suitability (*P* ≥ 0.6). Subsequently, the area of each suitability zone was calculated (Shi et al., 2023).

### 2.5 Spatial pattern change and centroid shift of medium and high suitability areas

To analyze the spatial pattern changes of medium and high suitability areas, the continuous suitability output from the MaxEnt model was converted into binary presence/absence maps in ArcGIS. Areas with a predicted probability (*P*) less than 0.4 were designated as unsuitable and assigned a value of 0, while areas with *P* ≥ 0.4 were considered potentially suitable and assigned a value of 1. This binary conversion was performed for each time period (current and future scenarios) to generate unsuitable/suitable binary data. Changes in suitability between periods were then categorized based on the binary values: 0–0 represents areas remaining unsuitable, 0–1 indicates newly gained suitable areas (expansion), 1–0 signifies lost suitable areas (contraction), and 1–1 denotes retained suitable areas (stable). The areas and geographic extent of expansion, retention, and contraction were calculated for *C. deserticola* under different climate scenarios relative to the current period.

Based on the binary suitable area maps, the geometric centroid of the potential suitable distribution for each period was calculated using the SDM toolbox. The changes in centroid location across different time periods were then compared to assess the overall shift trend of the core suitable area for *C. deserticola*, thereby reflecting the influence of environmental changes on its distribution.

### 2.6 Suitable habitat analysis under host plant constraint

Given the specific physiological characteristics of *C. deserticola*, its growth is obligately parasitic on the roots of *H. ammodendron* (Fan et al., 2023). To incorporate this host dependency into the habitat suitability modeling, a coupled “host suitability-environmental factor” modeling approach was developed. Initially, the potential suitable distribution of the host plant, *H. ammodendron*, was simulated using the optimized MaxEnt model (parameters: FC=LQHPT, RM=2.5). The output was a spatial distribution raster of continuous probability values ranging from 0 to 1 (asc format, 2.5′ resolution) (**Supplementary Figures S1**, **S2**). This *H. ammodendron* suitability raster was then utilized as a Spatially Explicit Constraint Layer. It was incorporated into the MaxEnt model for *C. deserticola* along with the selected six bioclimatic variables, five soil factors, and topographic data. During the model training process, the parasitic constraint was implemented through a Conditional Probability Transfer mechanism. Specifically, when the predicted suitability for *H. ammodendron* (P_HA_) fell below a defined threshold for effective parasitism (0.4, corresponding to medium and high suitability areas), the predicted suitability for C. deserticola (P_CD_) was forced to zero. Conversely, when P_HA_ was ≥ 0.4, P_CD_ was determined by the environmental variable response surfaces (Shao et al., 2022; Shen et al., 2019). The model was run 10 times to assess prediction stability.

To quantify the ecological effect of the host constraint, two contrasting scenarios were established: (1) Natural Habitat Scenario: *C. deserticola* distribution was predicted solely based on environmental variables. (2) Parasitic Constraint Scenario: Integrated prediction incorporating the *H. ammodendron* suitability constraint. The restrictive intensity of the host on *C. deserticola* distribution was analyzed by calculating the area difference rate of medium and high suitability areas (P ≥ 0.4) between the two scenarios. The area difference rate was calculated as: ((Area in Parasitic Constraint Scenario) - (Area in Natural Habitat Scenario))/(Area in Natural Habitat Scenario) × 100%. Additionally, changes in centroid location were assessed (Gong et al., 2023).

### 2.7 Land use analysis of stable suitable areas for *C. deserticola*

Land use data were obtained from the global land use prediction dataset for the period 2015–2100 (http://www.geosimulation.cn/Global-SSP-RCP-LUCC-Product.html). This dataset includes six land use types: Cropland, Forest, Grassland, Urban, Unused, and Water (Chen et al., 2022). The dataset exhibits high accuracy, with average Kappa coefficients, Overall Accuracy (OA), and Figure of Merit (FoM) values for global land simulation reported as 0.864, 0.929, and 0.102, respectively, and for the China region as 0.847, 0.886, and 0.137. This high accuracy both globally and within China indicates the reliability of the dataset, providing rich land use information beneficial for environmental impact assessment and future climate change studies (Zhao et al., 2021).

Spatial overlay analysis was performed to integrate the land use data with the potential stable suitable area distribution results simulated by the MaxEnt model. This analysis aimed to reveal the distribution characteristics of stable of *C. deserticola* suitable areas across different land use types (Liu et al., 2023). Furthermore, through spatial overlay analysis, the current and future potential suitable areas for *C. deserticola* predicted by the MaxEnt model were combined with land use data under different scenarios. This allowed for the calculation of suitable area extent and visualization of its change trends.

## 3 Result

### 3.1 Model accuracy evaluation and key environmental variables

The average test AUC values obtained were 0.947 ± 0.014 for *C. deserticola* under the Natural Habitat Scenario, 0.949 ± 0.016 for *C. deserticola* under the Parasitic Constraint Scenario, and 0.919 ± 0.017 for *H. ammodendron*. Notably, the average test AUC value in the Parasitic Constraint Scenario was slightly higher than that in the Natural Habitat Scenario. For future climate periods, both test and training AUC values consistently exceeded 0.9. These results indicate that the model performance achieved an “excellent prediction” level across all scenarios and time periods evaluated.

Variables with a cumulative contribution exceeding 85% are generally considered key drivers influencing species distribution (Yang et al., 2022). MaxEnt model predictions revealed the primary environmental variables shaping the geographic distribution of C. deserticola. In the Natural Habitat Scenario, the key variables were identified as prec7 (precipitation in July, with a suitable range of 5.8–74.5 mm, contributing 51.9%), ph_water (soil pH in water, with a suitable range of 7.3–10.8, contributing 13.8%), bio10 (mean temperature of the warmest quarter, with a suitable range of 13.6–28.0°C, contributing 13.3%), and prec10 (precipitation in October, with a suitable range of 0.8–27.8 mm, contributing 10.4%). These variables collectively accounted for a cumulative contribution of 89.4%.

In the Parasitic Constraint Scenario, the set of key variables influencing *C. deserticola* distribution shifted. The primary variables were prec7 (41.0%), P_HA_ (suitability probability of *H. ammodendron*, 26.3%), ph_water (9.9%), bio10 (8.9%), and prec10 (6.0%), with a cumulative contribution reaching 92.1% (**Supplementary Figure S3**). Compared to the Natural Habitat Scenario, the contribution of prec7 decreased from 51.9% to 41.0% but remained the most influential variable. P_HA_, representing the parasitic relationship, emerged as a significant factor, contributing 26.3% and becoming the second most important variable. Concurrently, the contributions of ph_water (9.9%), bio10 (8.9%), and prec10 (6.0%) were relatively reduced. These findings indicate that the introduction of the parasitic constraint enhanced the ability of model to explain the distribution of *C. deserticola* and underscored the significant role of the host-parasite relationship in shaping its geographic distribution pattern.

### 3.2 Current suitable habitat distribution under natural habitat and parasitic constraint scenarios

**Figure 4:**
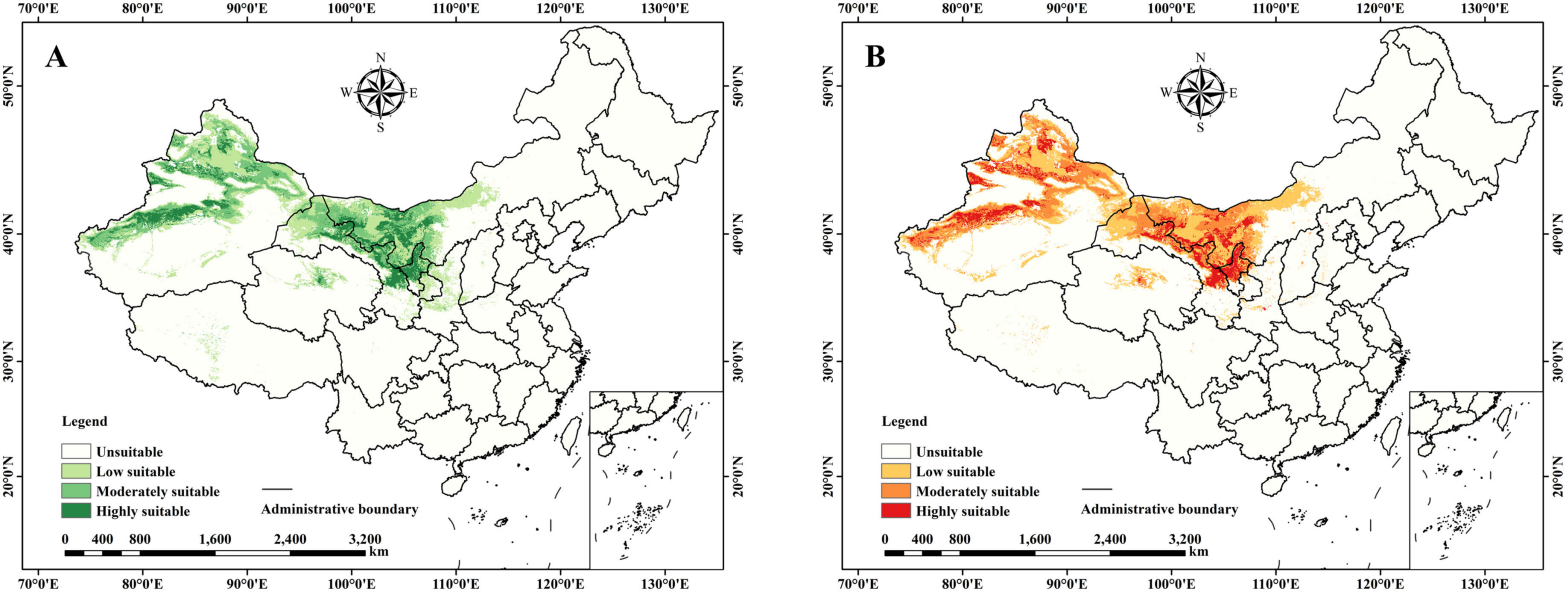
Current suitable area distribution of *C*. *deserticola*. **(A)** Natural Habitat Scenario. **(B)** Parasitic Constraint Scenario.

The current suitable habitat distribution of *C. deserticola* under the Natural Habitat and Parasitic Constraint Scenarios is presented in **Figure 4**. Significant differences in the distribution and extent of suitable habitat for *C. deserticola* were observed between the two constraint scenarios. Under the Natural Habitat Scenario (**Figure 4A**), the total suitable area for *C. deserticola* was estimated to be approximately 138.20 × 10^4^ km². This distribution was primarily concentrated in Northwest China, encompassing regions such as Xinjiang, Gansu, Qinghai, and western Inner Mongolia. Within this scenario, areas of high suitability (26.92 × 10^4^ km²) were predominantly located around the Tarim Basin and to the north of the Tianshan Mountains. Conversely, under the Parasitic Constraint Scenario (**Figure 4B**), the total suitable area was reduced to 131.92 × 10^4^ km², representing a decrease of approximately 6.28 × 10^4^ km². In this scenario, areas of high suitability were markedly concentrated in central Inner Mongolia and northern Gansu, indicating a clear limitation in the overall distribution range. This highlights the substantial impact of the parasitic constraint on the predicted suitable habitat and ecological range of *C. deserticola*.

### 3.3 Potential distribution and spatial pattern changes under future climate scenarios

Based on the MaxEnt model, integrated with host plant (*H. ammodendron*) distribution data, the potential suitable area distribution changes for *C. deserticola* were predicted under SSP126, SSP370, and SSP585 scenarios for the 2050s, 2070s, and 2090s (**Supplementary Figures S4**, **S5**, **Table 2**).

**Table 2:** Area of potential suitable habitat for *C. deserticola* (×10^4^ km²).

Scenario	Period	Natural habitat scenario			Parasitic constraint scenario		
		Highly suitable	Moderately suitable	Low suitable	Highly suitable	Moderately suitable	Low suitable
	current	26.92	47.19	64.09	23.88	46.02	62.02
SSP126	2050	31.31	42.43	59.32	26.07	40.98	56.65
	2070	31.86	43.23	57.20	29.10	41.79	58.59
	2090	37.14	41.52	58.19	37.14	37.95	56.88
SSP370	2050	35.86	43.68	61.08	30.88	40.48	58.66
	2070	52.16	45.33	60.95	40.59	42.20	63.96
	2090	53.82	42.67	62.48	48.22	41.27	62.22
SSP585	2050	40.64	41.19	57.75	33.36	41.04	57.75
	2070	49.61	42.72	63.54	40.23	44.38	60.39
	2090	52.39	45.16	63.60	48.00	43.72	60.76

Under future climate scenarios, the potential suitable area distribution of *C. deserticola* remained largely consistent with the current climate scenario, primarily located in the arid regions of Northwest China. This includes central and western Inner Mongolia, northern Gansu, southern Xinjiang, and the northeastern Qinghai-Tibet Plateau. Compared to the current period, the area of high suitability for *C. deserticola* showed an increasing trend in both the Natural Habitat and Parasitic Constraint Scenarios. Across both scenarios, the total suitable area generally decreased over time, while the area of high suitability significantly increased, and the area of low suitability continuously decreased. As shown in **Table 2**, the suitable area in the Parasitic Constraint Scenario was generally smaller than that in the Natural Habitat Scenario. For instance, under the SSP126 scenario in the 2050s, the total suitable area was 133.06 × 10^4^ km² in the Natural Habitat Scenario (a decrease of 3.7% compared to current) and 123.70 × 10^4^ km² in the Parasitic Constraint Scenario (a decrease of 6.2%). Under the SSP585 scenario in the 2090s, the high suitability area reached its maximum, measuring 52.39 × 10^4^ km² in the Natural Habitat Scenario (a 94.6% increase compared to current) and 48.00 × 10^4^ km² in the Parasitic Constraint Scenario (a 101.0% increase).

**Figure 5:**
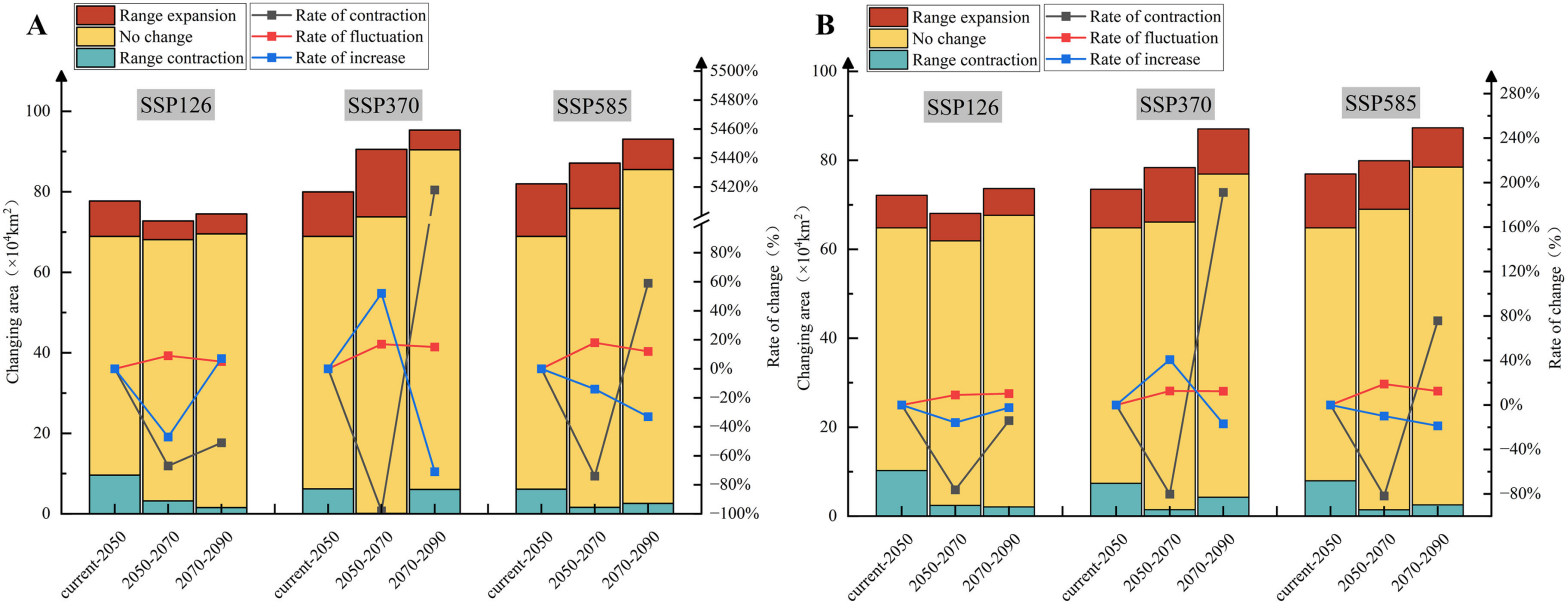
Spatial pattern changes in the potential suitable area of *C*. *deserticola*. **(A)** Natural Habitat Scenario. **(B)** Parasitic Constraint Scenario.

Spatial pattern changes in the medium and high suitability areas of *C. deserticola* were analyzed using binary MaxEnt model outputs (**Supplementary Figures S6**, **S7**, **Figure 5**). Results indicated that expansion areas for medium and high suitability were primarily concentrated in central, northeastern, and northwestern Inner Mongolia, northwestern Gansu, and northern, eastern, and central Xinjiang. The most significant expansion occurred under the SSP370 scenario between 2050 and 2070s, reaching 16.78 × 10^4^ km². Contraction areas for medium and high suitability were mainly distributed in northeastern Gansu, northern Xinjiang, and central Inner Mongolia. Notably, the contraction area reached 9.60 × 10^4^ km² under the SSP126 scenario from current to 2050s and 6.13 × 10^4^ km² under the SSP585 scenario from current to 2050s. Across different climate emission scenarios, contraction in medium and high suitability areas was particularly pronounced in northeastern Gansu, central Inner Mongolia, and large parts of Ningxia in the 2050s, generally exceeding that in other periods. It is worth noting that a relatively significant contraction area of 6.07 × 10^4^ km² was observed in the Inner Mongolia region under the SSP370 scenario between 2070 and 2090s. The spatial pattern changes of medium and high suitability areas under the Parasitic Constraint Scenario were similar to those under the Natural Habitat Scenario, but the areas were generally smaller than in the natural scenario. This reflects the regulatory role of host distribution in shaping the spatial pattern.

### 3.4 Centroid dynamics of *C. deserticola* suitable habitat

**Figure 6:**
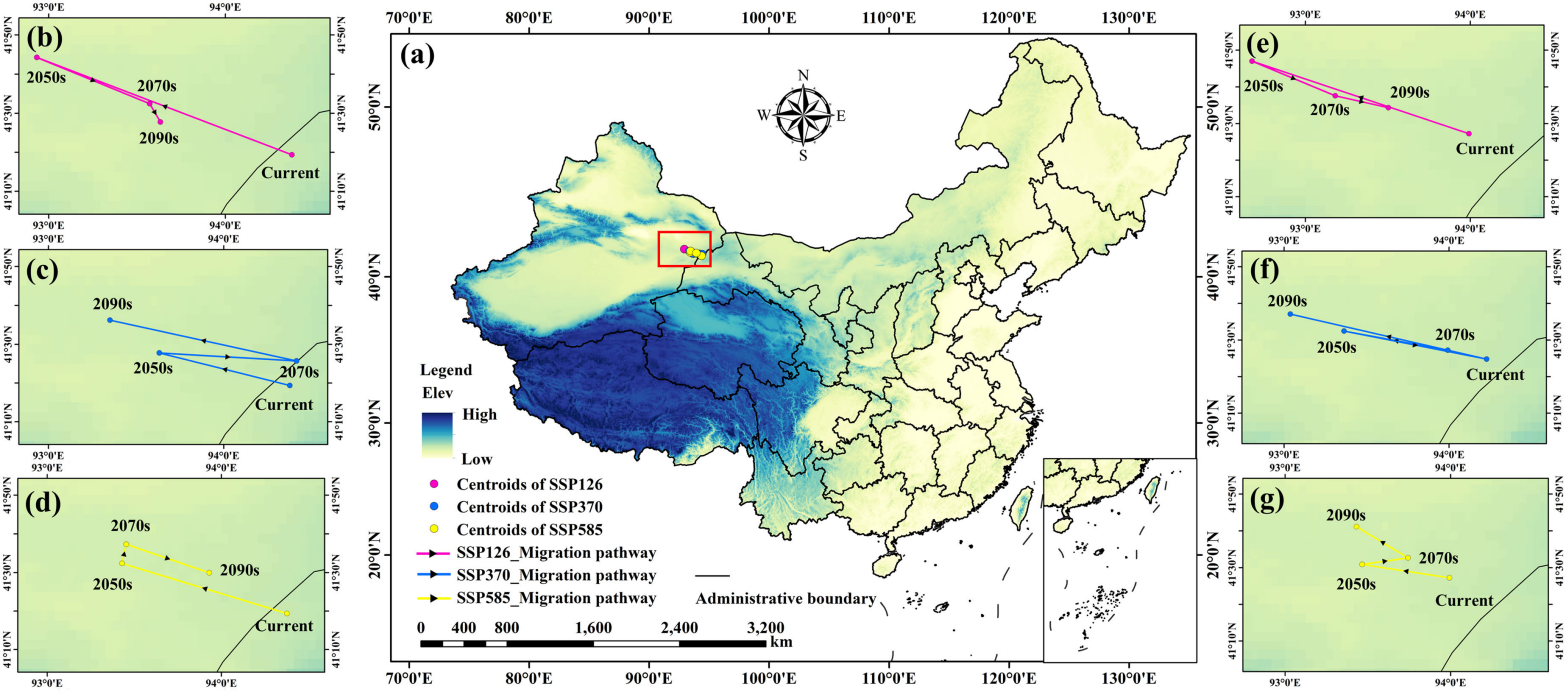
Centroid distribution of medium and high suitability areas for *C. deserticola* **(a)**. **(b-d)** Centroid changes under the Natural Habitat Scenario. **(e-g)** Centroid changes under the Parasitic Constraint Scenario.

The centroid locations and directional movement of the medium and high suitability areas for *C. deserticola* were calculated using ArcGIS software for the 2050s, 2070s, and 2090s under the SSP126, SSP370, and SSP585 scenarios (**Figure 6**). For the current period (1970–2000), the centroid of the suitable distribution was located in western Gansu (94.377°E, 41.323°N) under the Natural Habitat Scenario, and in eastern Xinjiang (93.992°E, 41.454°N) under the Parasitic Constraint Scenario.

Under future climate scenarios, the centroids of the medium and high suitability areas consistently shifted towards the northwest. The magnitude of centroid migration was generally greater in the Natural Habitat Scenario compared to the Parasitic Constraint Scenario. Cumulative migration distances varied across scenarios. The largest cumulative migration distance was observed under the SSP370 scenario (219.0 km in the Natural Habitat Scenario, 230.6 km in the Parasitic Constraint Scenario). Conversely, smaller migration distances were projected under the SSP585 scenario (153.6 km in the Natural Habitat Scenario, 97.0 km in the Parasitic Constraint Scenario).

The migration trajectories indicate that the suitable habitat for *C. deserticola* is projected to shift towards drier and colder conditions in the northwest, influenced by climate change. The smaller magnitude of centroid migration observed in the Parasitic Constraint Scenario suggests that host distribution limits the extent of this climate-driven shift. Furthermore, under similar climate conditions, the degree of centroid migration increased with increasing radiative forcing, both within the same time period and cumulatively over longer periods.

### 3.5 Land use analysis of *C. deserticola* suitable habitat

**Figure 7:**
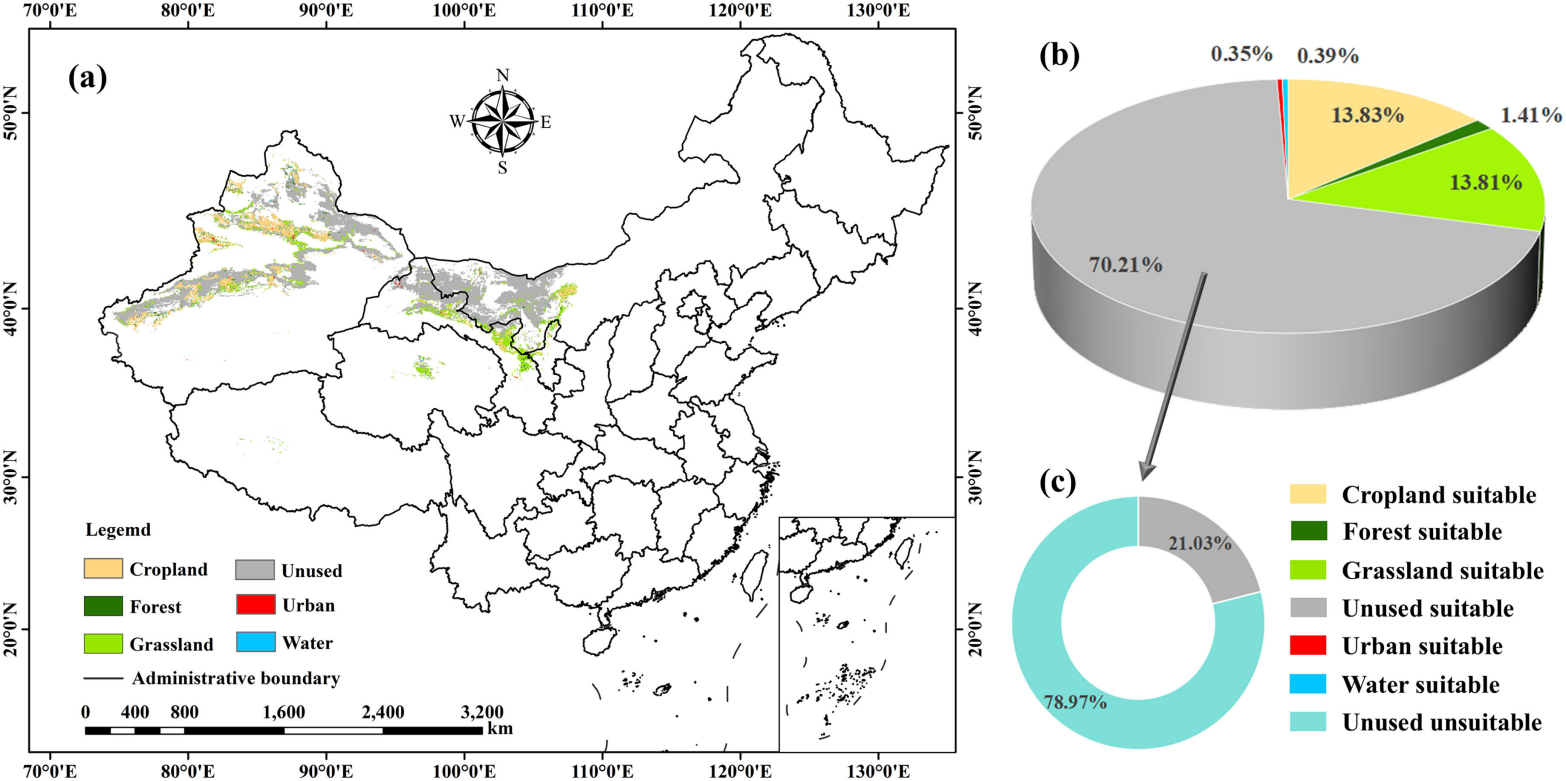
Land use characteristics within stable suitable areas of *C*. *deserticola*. **(a)** Land use types within stable suitable areas. **(b)** Proportion of land use types within suitable areas. **(c)** Proportion of Unused land within suitable areas relative to total Unused land.

The MaxEnt output of suitable area distribution (current and future) was reclassified into two categories based on the suitability index: suitable areas (0.4–1) and unsuitable areas (0–0.4). These were then assigned binary values of 1 (suitable) and 0 (unsuitable), respectively. Spatial overlay analysis was performed on the reclassified binary maps from different periods using the Raster Calculator tool in ArcGIS. The resulting overlapping areas were identified as stable suitable areas for *C. deserticola*. These stable suitable areas were then overlaid with land use type data to analyze the land use characteristics within them (**Figure 7**).

Results indicated that the stable suitable areas for *C. deserticola* are primarily distributed in Northwest China and parts of Inner Mongolia, exhibiting a diversity of land use types (**Figure 7a**). Based on the land use classification, the dominant types within these suitable areas include Unused land, Grassland, Cropland, Forest, Urban, and Water. Quantification of land use proportions within the stable suitable areas (**Figure 7b**) revealed that Unused land accounted for the largest proportion, reaching 70.21%, indicating its absolute dominance in terms of area. Grassland and Cropland represented significant proportions as well, at 13.81% and 13.83%, respectively, covering areas of approximately 90,298.54 km² and 90,447.17 km². These were the other major land use types within the stable suitable areas. Further analysis of the proportion of Unused land within the suitable areas relative to the total Unused land (**Figure 7c**) showed that Unused land within the *C. deserticola* suitable areas constituted 21.03% of the total Unused land, covering an area of 459,072.64 km².

**Figure 8:**
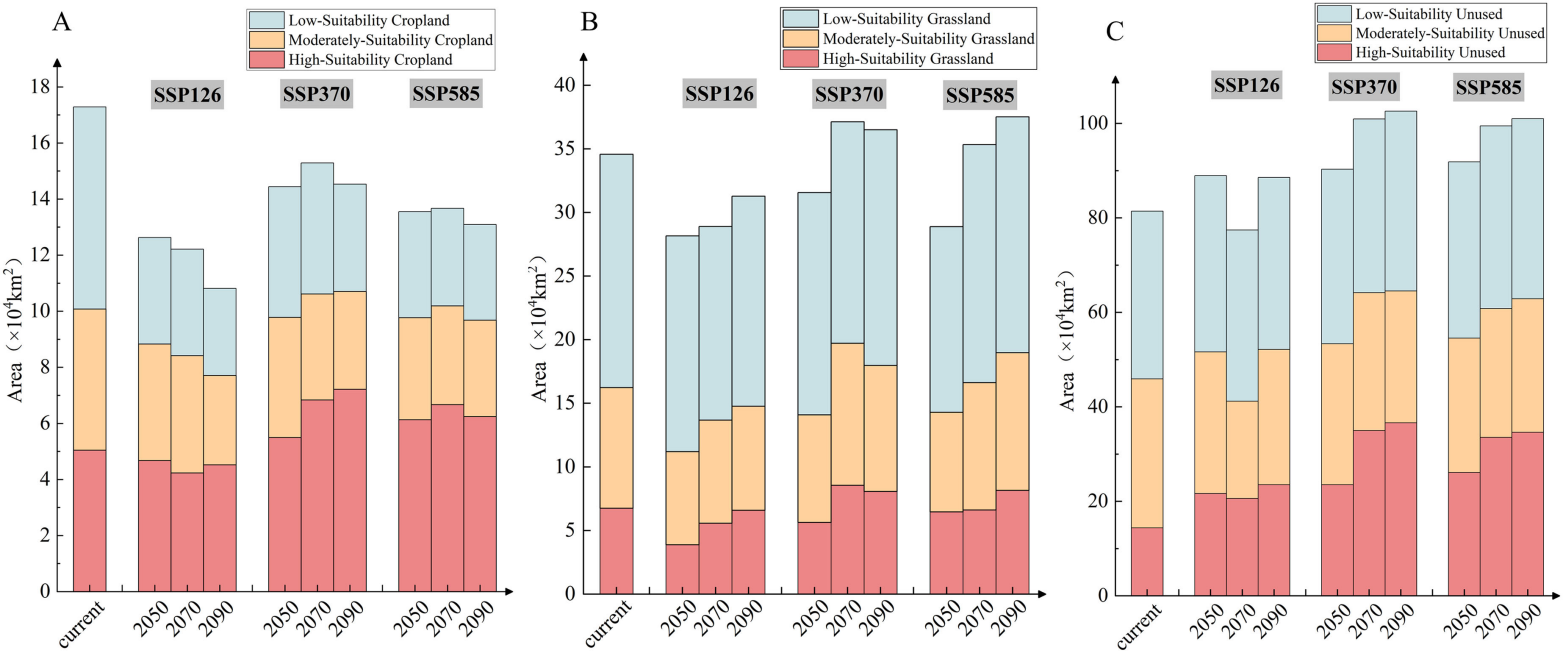
Potential suitable areas of *C*. *deserticola* by land use type. **(A)** Suitable areas within Cropland. **(B)** Suitable areas within Grassland. **(C)** Suitable areas within Unused land.

Based on the land use analysis of stable suitable areas, the primary land use types within *C. deserticola* suitable habitat were identified as Unused land (459,072.64 km²), Grassland (90,298.54 km²), and Cropland (90,447.17 km²). Consequently, spatial overlay was performed to examine the potential suitable area distribution patterns within these three land use types under current and future scenarios. Considering the ecological requirements of *C. deserticola* and its suitable environment, coupled with China’s cultivated land protection policy, Cropland suitable areas were excluded from further analysis (**Supplementary Figure S8**, **Figure 8A**). The focus was placed on analyzing the spatial distribution patterns of suitable areas within Unused land and Grassland, identifying these as core regions for potential *C. deserticola* cultivation.

Results indicated that suitable areas within Unused land remained dominant across all climate scenarios, with their area continuously increasing (**Figure 8C**). Their spatial distribution was primarily concentrated in northern Xinjiang, western Gansu, and western Inner Mongolia (**Supplementary Figure S9**). These regions are characterized by Gobi deserts, arid lands, and desertification margins, which highly align with the ecological characteristics of *C. deserticola* parasitizing desert plants (e.g., *H. ammodendron*), making them the most suitable areas for cultivation. Suitable areas within Grassland also showed an increasing trend under future scenarios (**Figure 8B**), with their distribution gradually expanding towards northeastern Gansu and central-eastern Inner Mongolia (**Supplementary Figure S10**). These grasslands are often semi-arid or desert grasslands located at the margins of land desertification, suitable for the growth of *C. deserticola*. Therefore, future cultivation planning for *C. deserticola* should focus on suitable areas within Unused land and Grassland. This strategy aligns not only with the ecological requirements of *C. deserticola* but also with China’s cultivated land protection policy. By maximizing its ecological value, this approach can simultaneously strengthen the material foundation for traditional Chinese medicine.

## 4 Discussion

### 4.1 Influence of key environmental variables on *C. deserticola* distribution

Evaluation of key environmental variables revealed that the current suitable habitat distribution of *C. deserticola* is primarily limited by four dominant factors: precipitation in July (prec7, 5.8–74.5 mm), soil pH (ph_water, 7.3–10.8), mean temperature of the warmest quarter (bio10, 13.6–28.0°C), and precipitation in October (prec10, 0.8–27.8 mm). When considered in conjunction with the parasitic constraint, these factors further refine the limiting conditions of the *C. deserticola* ecological niche.

Precipitation and temperature are recognized as core drivers of plant growth, and their synergistic effects are particularly pronounced in arid regions (Fang et al., 2024).Specifically, precipitation in July (prec7) provides essential moisture support during the vegetative growth phase of *C. deserticola*. However, excessive rainfall during this period can potentially induce root hypoxia or promote the proliferation of pathogens, consequently inhibiting growth (Chen et al., 2024). The identified strict lower limit for October precipitation (prec10) is likely linked to the tolerance of *C. deserticola* to water stress during its initial parasitic establishment. Conversely, excessively high autumn precipitation might disrupt nutrient translocation from the host *H. ammodendron* by altering soil moisture dynamics (Fan et al., 2023). Regarding temperature, the mean temperature of the warmest quarter (bio10) appears to reflect the high-temperature tolerance of *C. deserticola* during its period of metabolic activity (Miao et al., 2023a).

The high threshold identified for soil pH (ph_water) underscores the strong dependence of *C. deserticola* on alkaline soil conditions. This specific characteristic is highly consistent with the ecological adaptation strategies of its host, *H. ammodendron* (Hu et al., 2021). The adaptation of *C. deserticola* to high pH environments suggests the presence of specialized physiological mechanisms for coping with alkaline stress. These mechanisms are likely closely linked to the regulation of host of the rhizosphere microenvironment. *H. ammodendron*, possessing a well-developed deep root system, frequently accesses groundwater, which can lead to an increase in rhizosphere soil pH, thereby creating a high-alkaline ecological niche (Hu et al., 2021). Furthermore, alkaline soil conditions may enhance the specificity of the parasitic interaction between *C. deserticola* and *H. ammodendron* by suppressing non-adapted microbial communities and reducing competition from other plant species (Zhang et al., 2016).

The natural parasitic success rate of *C. deserticola* on *H. ammodendron* is typically low, often reported below 30%. This is primarily limited by the availability of suitable host individuals and the precise matching of environmental conditions required for successful establishment. However, through targeted interventions such as genetic screening, microbial regulation, and artificial inoculation techniques, the parasitic success rate can be significantly enhanced, potentially reaching 50–70% (Shen et al., 2019). In this study, the adoption of a host suitability probability threshold (≥0.4) as a parasitic constraint aimed to reflect both the ecological reality of host dependency and the potential for successful parasitism under favorable or optimized conditions.

Under the Parasitic Constraint Scenario, the host suitability factor (P_HA_) exhibited a substantial contribution of 26.3%, ranking second only to July precipitation. This finding strongly indicates that the distribution of *H. ammodendron* is a critical determinant shaping the geographic pattern of *C. deserticola*. Particularly in arid environments, the presence of a suitable host is a prerequisite for the survival and establishment of *C. deserticola*.

### 4.2 Spatial pattern analysis of potential suitable areas under two scenarios

Comparison of the Natural Habitat Scenario (NHS) and the Parasitic Constraint Scenario (PCS) revealed a notable reduction in the extent of potential suitable area for *C. deserticola* under PCS, decreasing by 4.5% (from 138.20 × 10^4^ km² to 131.92 × 10^4^ km²). This finding underscores the significant limiting effect of the host plant distribution of *H. ammodendron* on the overall distribution of *C. deserticola*. In terms of spatial distribution, suitable areas under PCS were more concentrated in central Inner Mongolia and northern Gansu, while also exhibiting a more fragmented pattern.

This marked reduction under the PCS, particularly evident in the fragmentation of suitable areas, carries significant implications for conservation. Such a contraction and isolation of potential habitat patches can substantially threaten the long-term persistence of *C. deserticola* populations. Reduced habitat size and increased fragmentation can lead to smaller, more isolated subpopulations, making them more susceptible to genetic drift, inbreeding, environmental stochasticity, and reduced dispersal and gene flow. This vulnerability is heightened by the obligate parasitic dependency; the parasite population is limited not only by its own environmental needs but also by the spatial availability and environmental suitability of its host. Therefore, the insights derived from the PCS are critical for identifying genuinely viable habitat areas and designing targeted conservation strategies that account for both environmental limits and the spatial distribution and health of the host.

Further analysis incorporating future climate projections (SSP126, SSP370, SSP585 for the 2050s, 2070s, and 2090s), specifically examining area difference rates and centroid migration distances, provided insights into the dynamic influence of host availability on distribution patterns. **Supplementary Figure S11** illustrates that under the current scenario, the area difference rate for high suitability areas was -11.30%, and for medium suitability areas, it was -2.48%. In future scenarios, the area difference rate for high suitability areas decreased further, reaching -16.74% under SSP126-2050s and -18.92% under SSP585-2070s. This trend indicates that the parasitic constraint exacerbates the reduction in suitable area extent over time.

Centroid migration analysis showed that the centroid of suitable areas under PCS consistently shifted towards the north or northwest. This shift was particularly pronounced under high emission scenarios such as SSP585, reflecting the combined effects of climate change and the spatial heterogeneity of host distribution. These findings are consistent with previous research (He et al., 2021). The significant shaping effect of the parasitic constraint on the suitable area pattern was further supported by the higher AUC value of the PCS model compared to the NHS, validating its enhanced ecological realism. Furthermore, the observed future area difference rates (e.g., -18.92% for high suitability areas under SSP585-2070s) align with predictions of suitable area migration towards higher latitudes (Liu et al., 2019), suggesting that climate change may intensify the contraction of suitable habitats. The trend towards fragmentation and centroid shift observed in the PCS results also corroborates the concept of parasite-host spatial mismatch proposed by Lu et al. (2022), implying that dynamic changes in host distribution could further exacerbate the differentiation of *C. deserticola* suitable areas. The analysis of area difference rates and centroid migration provides a quantitative understanding of the intensity of host limitation. The significantly higher area difference rate for current high suitability areas (-11.30%) compared to medium suitability areas (-2.48%) indicates that the parasitic constraint has a more pronounced impact on highly suitable habitats. This trend is further amplified in future projections, as seen in the -18.92% difference rate for high suitability areas under SSP585-2070s. Conservation and cultivation strategies should therefore prioritize the current and future high suitability areas identified by the PCS (e.g., central Inner Mongolia). Mitigating host limitation through optimizing parasitic efficiency via artificial cultivation techniques represents a crucial approach to support the sustainable management of *C. deserticola*.

### 4.3 Influence of land use on *C. deserticola* distribution patterns

The results indicate that Unused land, particularly in desertified regions, provides crucial habitat for *C. deserticola* due to its parasitic relationship with *H. ammodendron*. This habitat is essential for enhancing the ecological functions associated with *C. deserticola* and its host, including promoting soil stability, increasing vegetation cover, and maintaining the balance of arid ecosystems (Li et al., 2012; Shen et al., 2019). Furthermore, Grassland, identified as a secondary suitable area, also plays a significant role in the distribution of *C. deserticola*. However, future urbanization and cropland expansion pose a potential threat, which may lead to the fragmentation and reduction of suitable areas within grasslands. Addressing this challenge requires careful land-use planning that effectively reconciles the protection of these critical uncultivated areas with increasing development pressures to ensure the sustainable utilization and conservation of this species.

Compared to other studies, this research offers a novel perspective on the influence of land use on species distribution. For instance, Liu et al. (2023) found that land use types significantly impact the distribution of tea oil tree species, which aligns with the general finding of this study regarding the importance of land use. However, this study further reveals the dominant role of Unused land and Grassland in the distribution of *C. deserticola*. This is in stark contrast to the pattern observed for tea oil tree species, which typically prefer forest and shrubland habitats. This difference reflects the unique adaptation of *C. deserticola* to arid and semi-arid ecosystems.

Under future climate scenarios, the area of suitable habitat within both Unused land and Grassland is projected to increase. Specifically, under the SSP585–2090 scenario, the total suitable area within Unused land is projected to reach 101.02 × 10^4^ km², with a significant increase in the proportion of highly suitable areas. Concurrently, suitable areas within Grassland are projected to expand towards northeastern Gansu and central-eastern Inner Mongolia. These trends suggest that climate warming may indirectly expand desertified areas by exacerbating aridity, thereby providing more suitable habitat for *C. deserticola* (Liu et al., 2019).

To navigate the complexities of future land use changes and development pressures, our findings highlight the necessity of integrated land-use planning approaches. First and foremost, prioritizing the conservation of suitable habitat areas identified within Unused land and Grassland is paramount. This involves establishing targeted protection zones or incorporating these areas into existing conservation frameworks. Second, implementing spatial planning tools can help designate areas for strict protection versus areas where sustainable utilization, such as low-intensity artificial cultivation of *C. deserticola* integrated with local agricultural and pastoral practices, can be promoted. Drawing lessons from conservation strategies for other species, such as *Sterculia villosa* (Luo et al., 2024), dynamic monitoring and ecological compensation mechanisms could be implemented to mitigate the encroachment of urbanization and other development activities on suitable habitats. Furthermore, maintaining and enhancing landscape connectivity through ecological corridors is crucial to counteract habitat fragmentation caused by land use change and support species dispersal and gene flow.

### 4.4 Resource-ecology-economy sustainable development in arid and semi-arid regions

**Figure 9:**
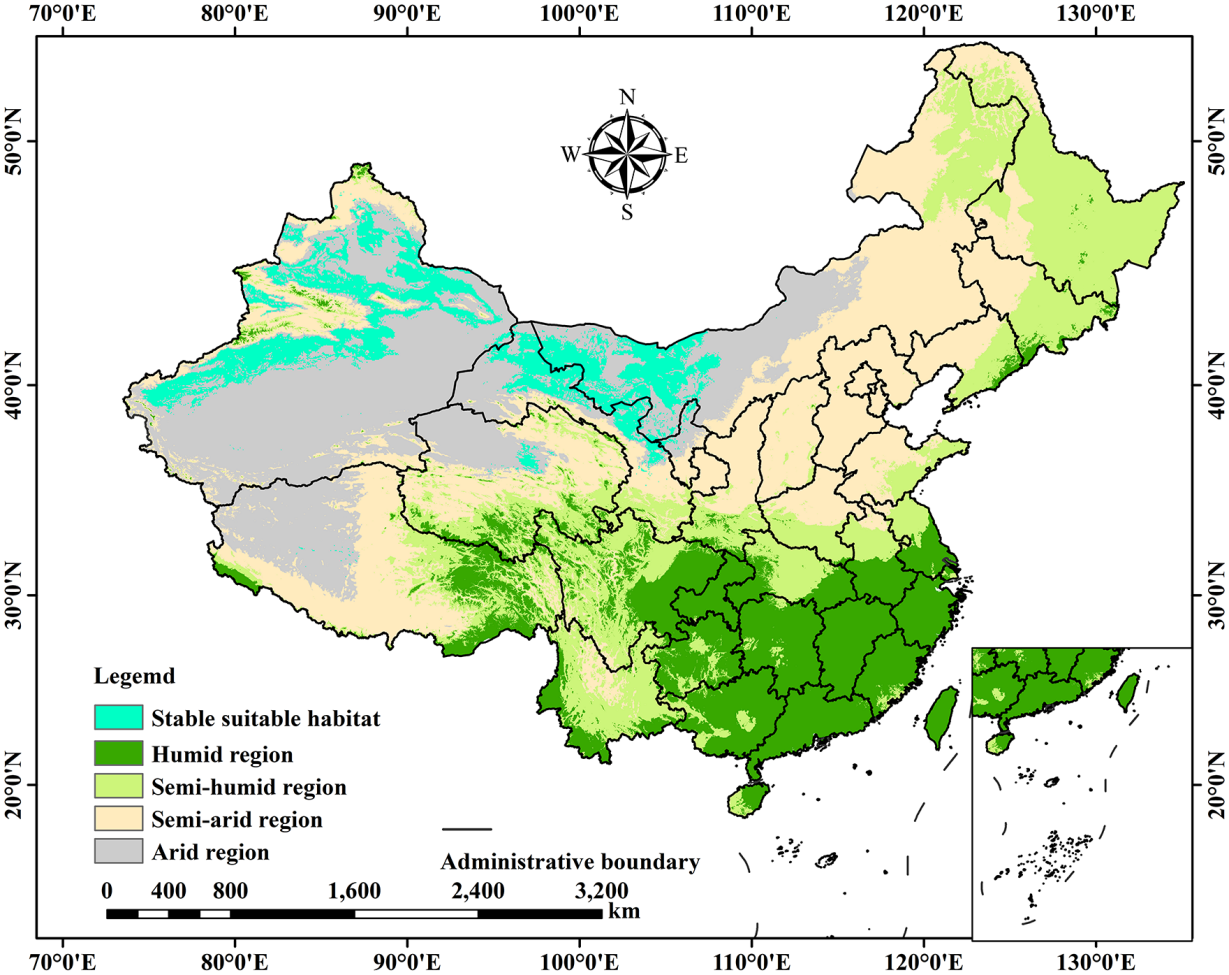
Distribution of stable suitable areas for *C. deserticola* within the dry and wet zones of China.

Arid and semi-arid regions face multiple challenges stemming from resource scarcity, ecological fragility, and economic backwardness. Achieving sustainable development that integrates resources, ecology, and economy is a core objective for regional progress (Yan et al., 2024). This study contributes to this goal by analyzing the distribution pattern of *C. deserticola* and its relationship with land use types. By linking these findings to the dry and wet zone map of China (**Figure 9**), it is revealed that the stable suitable areas for *C. deserticola* are primarily concentrated in arid regions (e.g., Xinjiang, western Gansu) and the margins of northwestern semi-arid areas (e.g., central-western Inner Mongolia, northeastern Gansu), with Unused land (70.21%) and Grassland (13.81%) serving as the main habitats. This provides a scientific basis for promoting sustainable regional development.

Reconciling the protection of these crucial natural habitats with economic development pressures requires a multi-faceted strategy embedded within regional land-use planning. As a valuable traditional Chinese medicine, the symbiotic system of *C. deserticola* and its host *H. ammodendroncan* enhance vegetation cover, increase soil organic matter, and bolster ecosystem resilience. Under appropriate planning, low-intensity artificial cultivation integrated with local agriculture and pastoralism can facilitate the sustainable utilization of resources and promote income growth for local communities, provided it is conducted in designated suitable areas and does not lead to the degradation of critical natural habitats. Protecting these critical Unused land and Grassland areas is paramount. By establishing ecological corridors to enhance landscape connectivity, the fragmentation of suitable areas can be effectively mitigated, and the expansion of desertification can be curbed (Wu and Convertino, 2025). Leveraging ecological compensation mechanisms can further incentivize community participation, promoting ecological restoration and biodiversity maintenance.

Furthermore, economic development in arid regions often lags behind, leading to pronounced conflicts between resource exploitation and ecological conservation. Developing the *C. deserticola* industry through symbiotic cultivation on unused land offers a pathway that not only safeguards the ecosystem but also stimulates related sectors such as traditional Chinese medicine processing and eco-tourism, thereby maximizing economic benefits. Integrating this with land use policies to optimize cultivation layout can foster the development of an eco-economic belt along the arid margins, thereby harmonizing resource utilization, ecological protection, and economic sustainability, and providing practical guidance for regional development.

## 5 Conclusion

This study employed an optimized MaxEnt model, integrating parasitic constraints and land use dynamics, to predict the potential suitable habitat for *C. deserticola* under current and future climate scenarios. By incorporating the suitability probability of the host plant (*H. ammodendron*) as a continuous probability constraint and overlaying high-resolution land use data, the ecological realism of the MaxEnt predictions was significantly enhanced. This was evidenced by the achieved AUC values of 0.947 ± 0.014 for the Natural Habitat Scenario and 0.949 ± 0.016 for the Parasitic Constraint Scenario. The results demonstrate that the parasitic constraint led to a 4.5% reduction in suitable habitat area (from 138.20 × 10^4^ km² to 131.92 × 10^4^ km²) and resulted in a more fragmented distribution, particularly in Northwest China (e.g., Inner Mongolia and Gansu). Predictions under future SSP126, SSP370, and SSP585 scenarios indicated an overall decrease in total suitable habitat area, but a significant increase in the area of highly suitable regions. Concurrently, the centroid of suitable habitat is projected to shift towards drier and colder areas in the northwest. Land use analysis revealed that Unused land (70.21%) and Grassland (13.81%) constitute the primary habitats for *C. deserticola*, highlighting their critical role in sustainable cultivation strategies. These findings underscore the importance of both host availability and land use dynamics in shaping the distribution of this parasitic plant. The results provide a solid scientific basis for developing adaptive management strategies that balance ecological restoration, medicinal resource utilization, and sustainable development in arid and semi-arid regions.

## Data Availability

The original contributions presented in the study are included in the article/**Supplementary Material**. Further inquiries can be directed to the corresponding author.
