# Supplementary material for: Predicting potential suitable habitat of Cistanche deserticola by integrating parasitic constraints and land use data into MaxEnt modeling
Source: Front Plant Sci. 2025 Aug 4;16:1635595. doi: 10.3389/fpls.2025.1635595 (PMC12358397; doi:10.3389/fpls.2025.1635595)
Supplement: Supplementary file 1 [file DataSheet1.pdf]

Table S1.Environmental variables selected for modeling（a：*C. deserticola*，b：*H. ammodendron*）

Variable code	Variable name	Unit	species
bio10	Mean temperature of the warmest quarter	℃	a
prec10	Precipitation in October	mm	a
bio11	Mean temperature of the coldest quarter	℃	a
prec12	Precipitation in December	mm	a
prec7	Precipitation in July	mm	a、b
ph_water	Soil pH	-	a、b
bio15	Precipitation seasonality (Coefficient of variation)	mm	a、b
coarse	Soil sand grain size	%	a、b
slope	Slope gradient	°	a、b
sand	Sand content	%	a、b
aspect	Slope aspect	-	a、b
bio4	Standard deviation of seasonal variation of temperature	-	b
prec11	Precipitation in November	mm	b
elev	Altitude	m	b
tmin10	Minimum temperature in October	℃	b
org_carbon	Organic carbon content	%	b

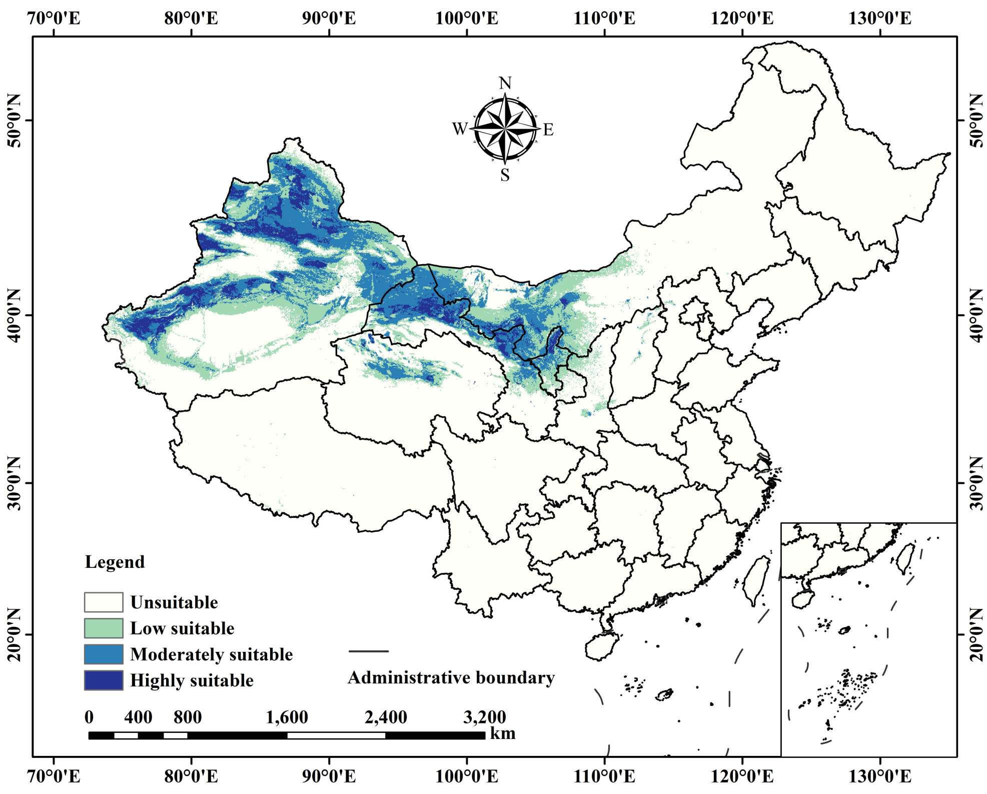

Fig S1. Current Distribution of Suitable Areas for *H. ammodendron*

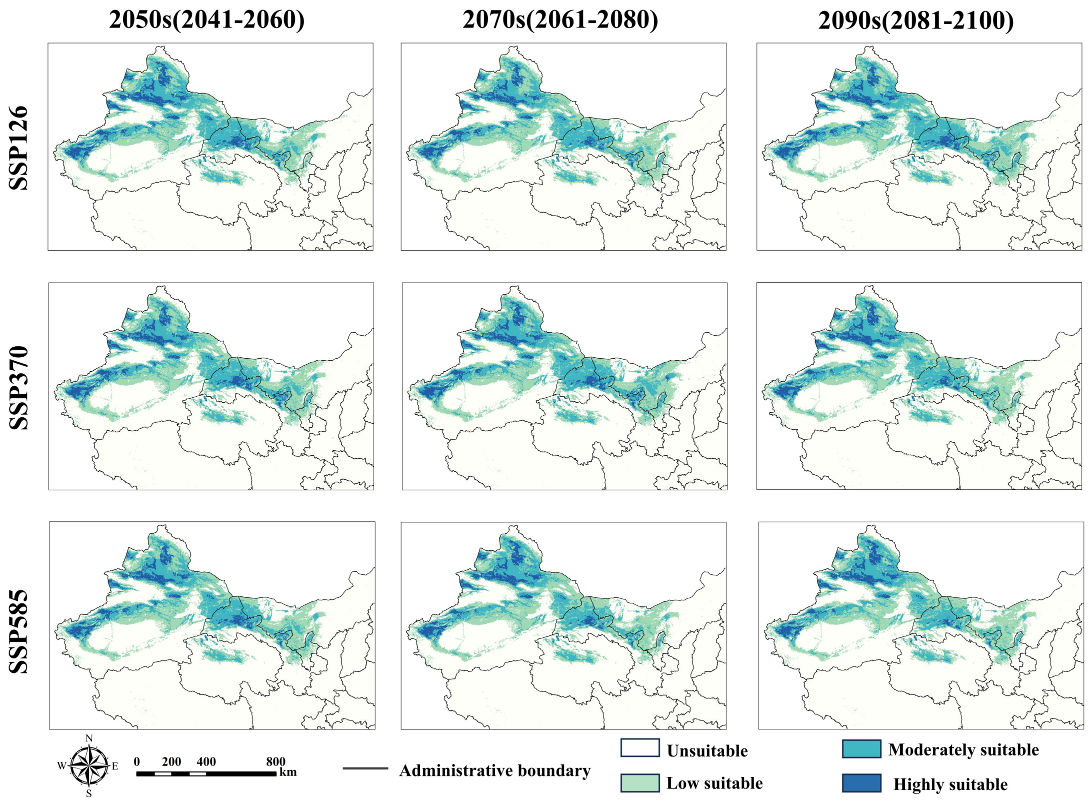

Fig S2. Potential Suitable Area Distribution for *H. ammodendron*

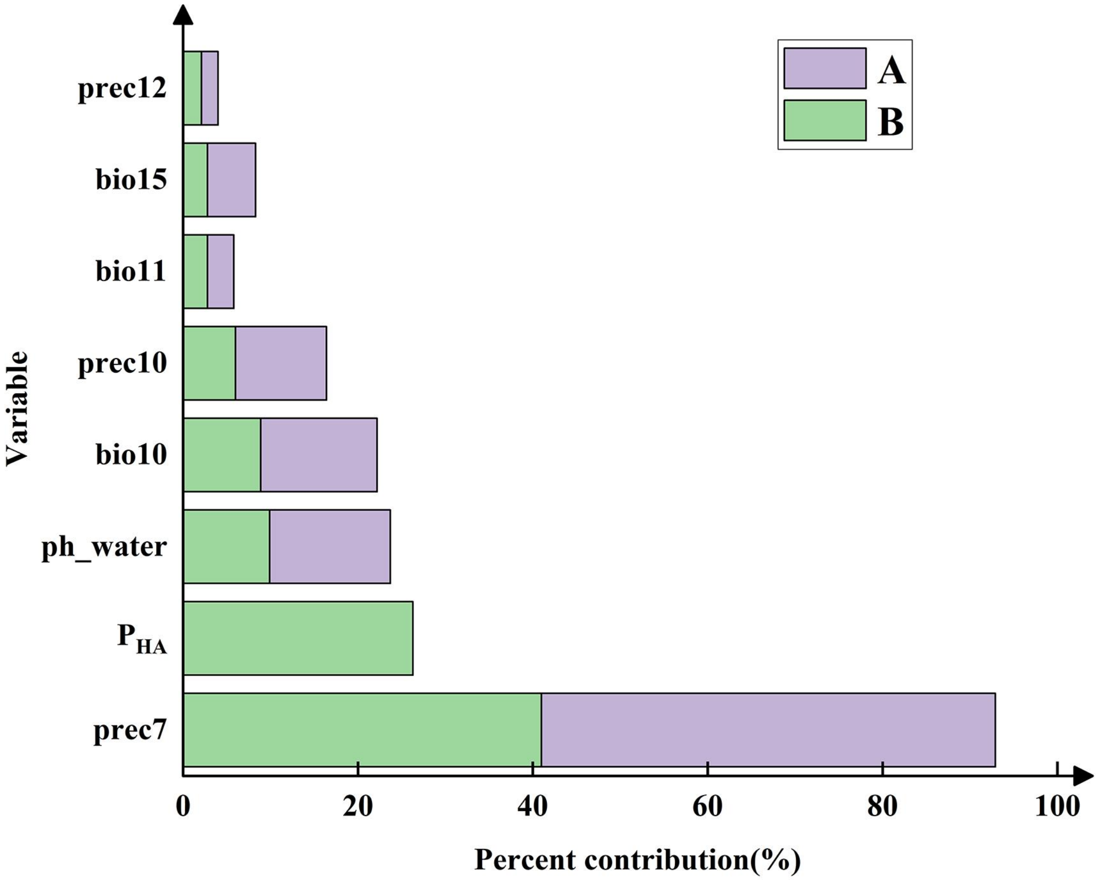

Fig S3. Contribution of environmental variables in the MaxEnt model. (A) Contribution under the Natural Habitat Scenario. (B) Contribution under the Parasitic Constraint Scenario

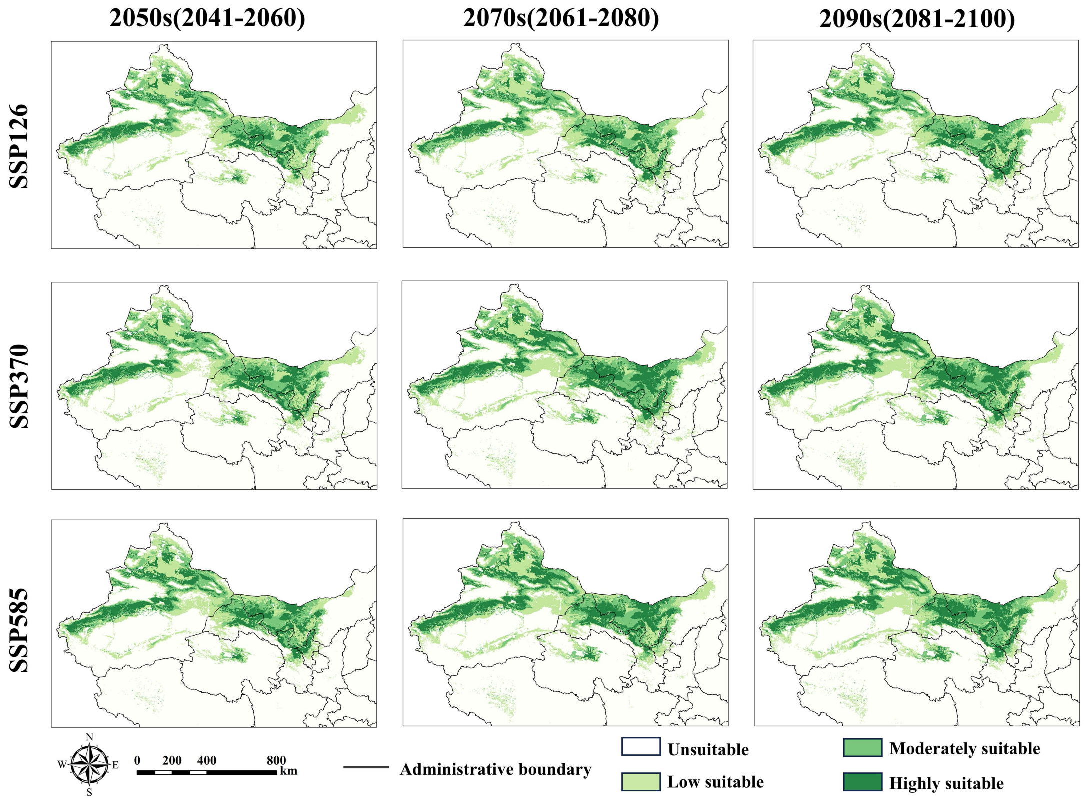

Fig S4. Potential suitable area distribution of C. deserticola under the Natural Habitat Scenario

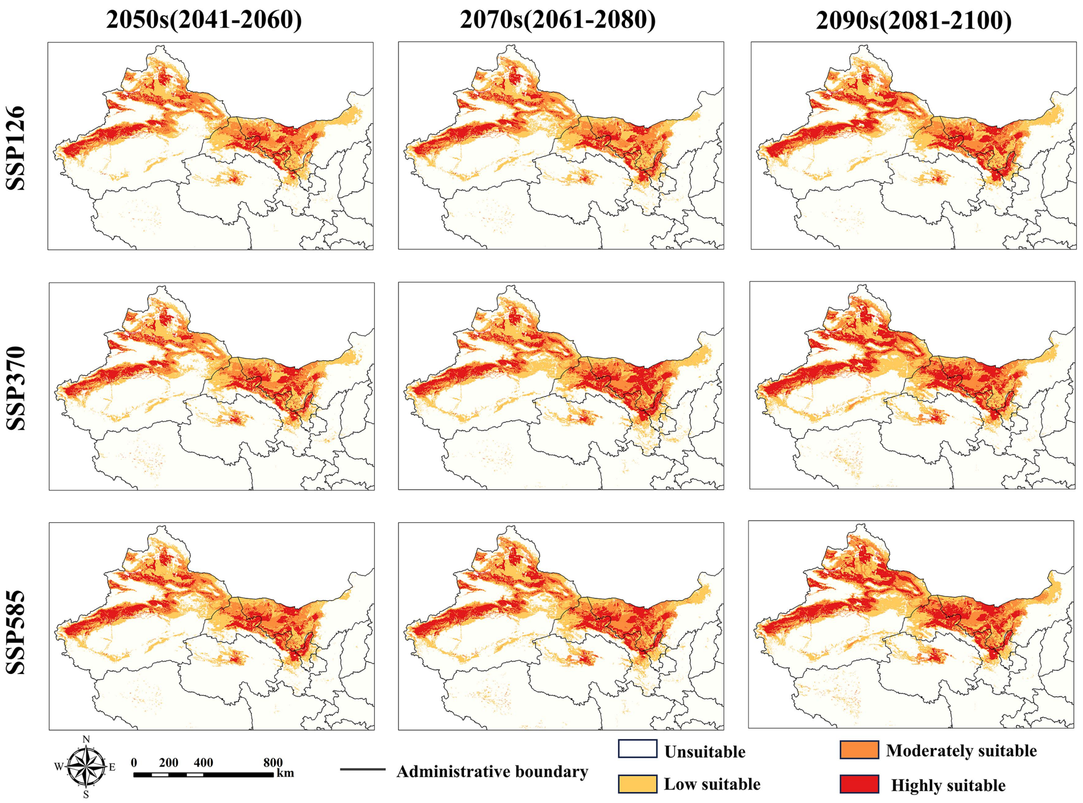

Fig S5. Potential suitable area distribution of C. deserticola under the Parasitic Constraint Scenario

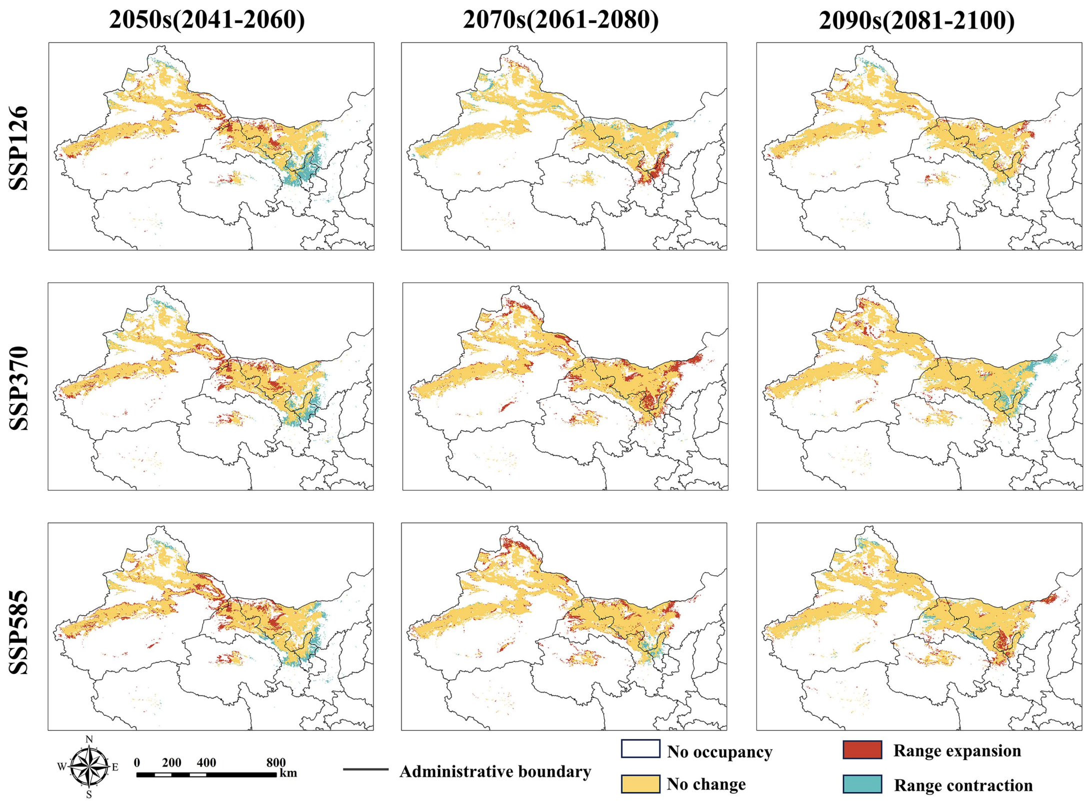

Fig S6. Changes in potential suitable area over time under the Natural Habitat Scenario

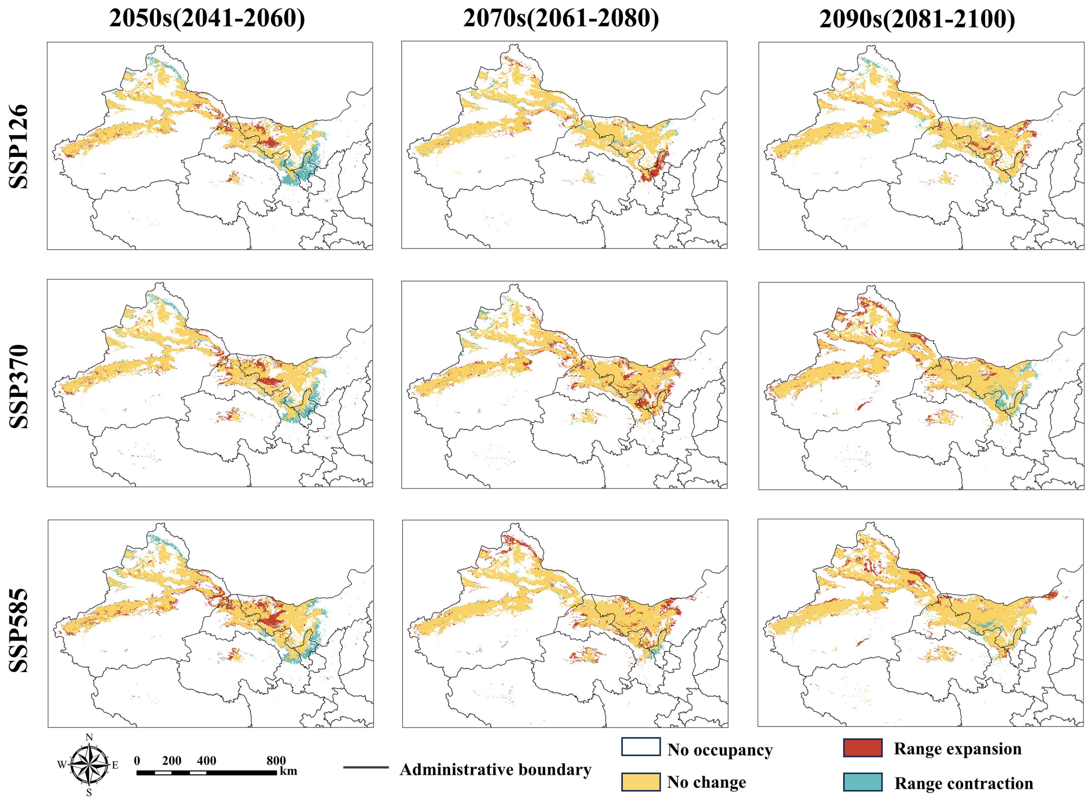

Fig S7. Changes in potential suitable area over time under the Parasitic Constraint Scenario

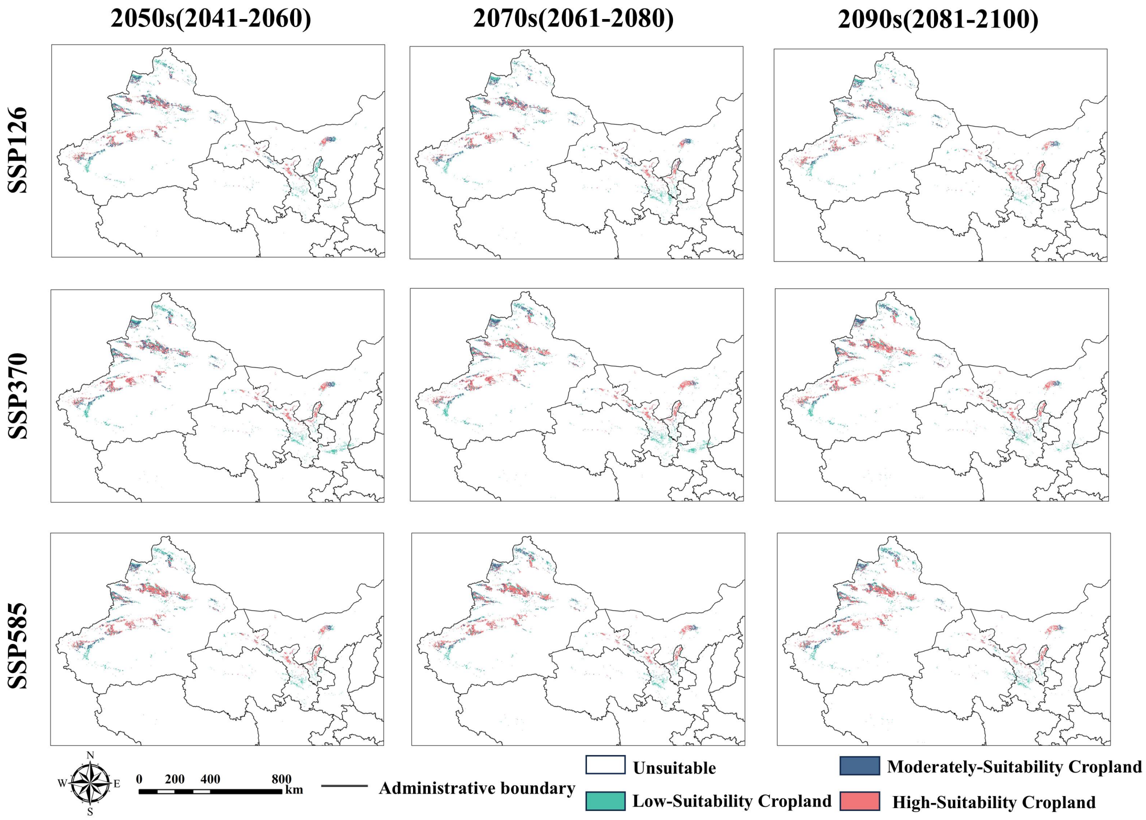

Fig S8. Distribution of Suitable Areas for *C. deserticola* in Cropland

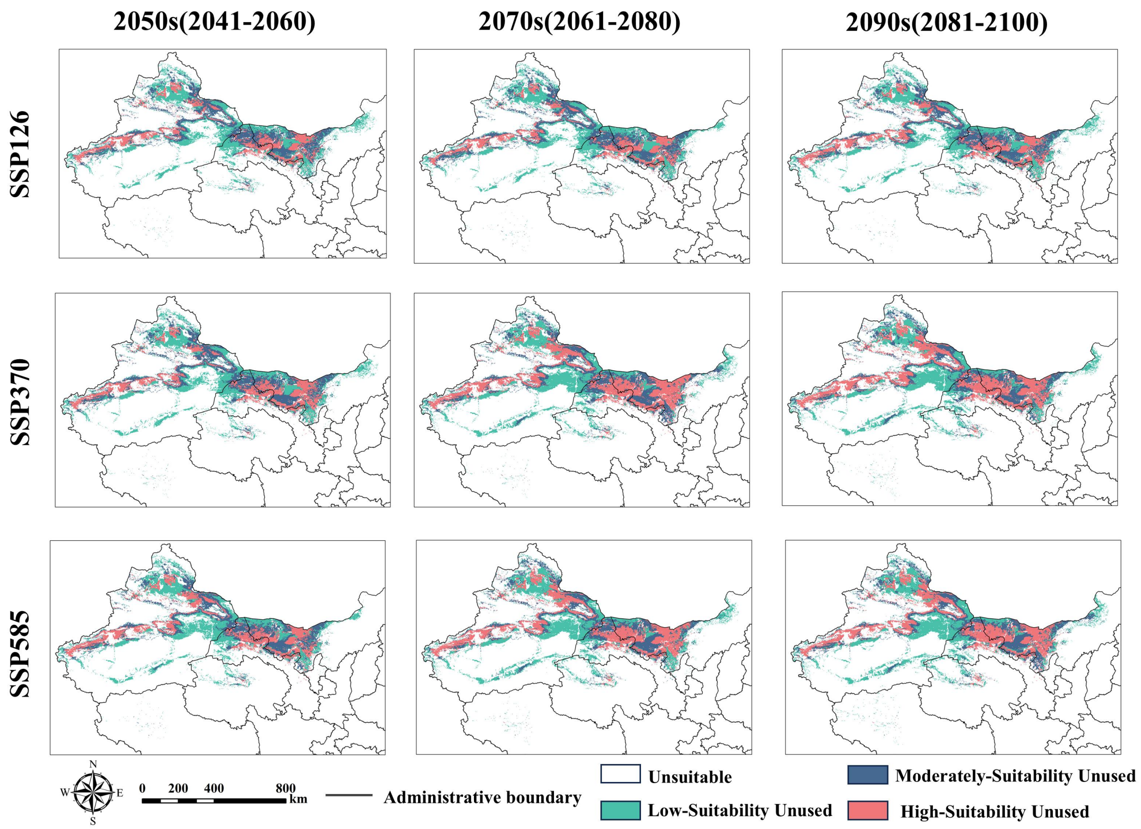

Fig S9. Distribution of Suitable Areas for *C. deserticola* in Unused

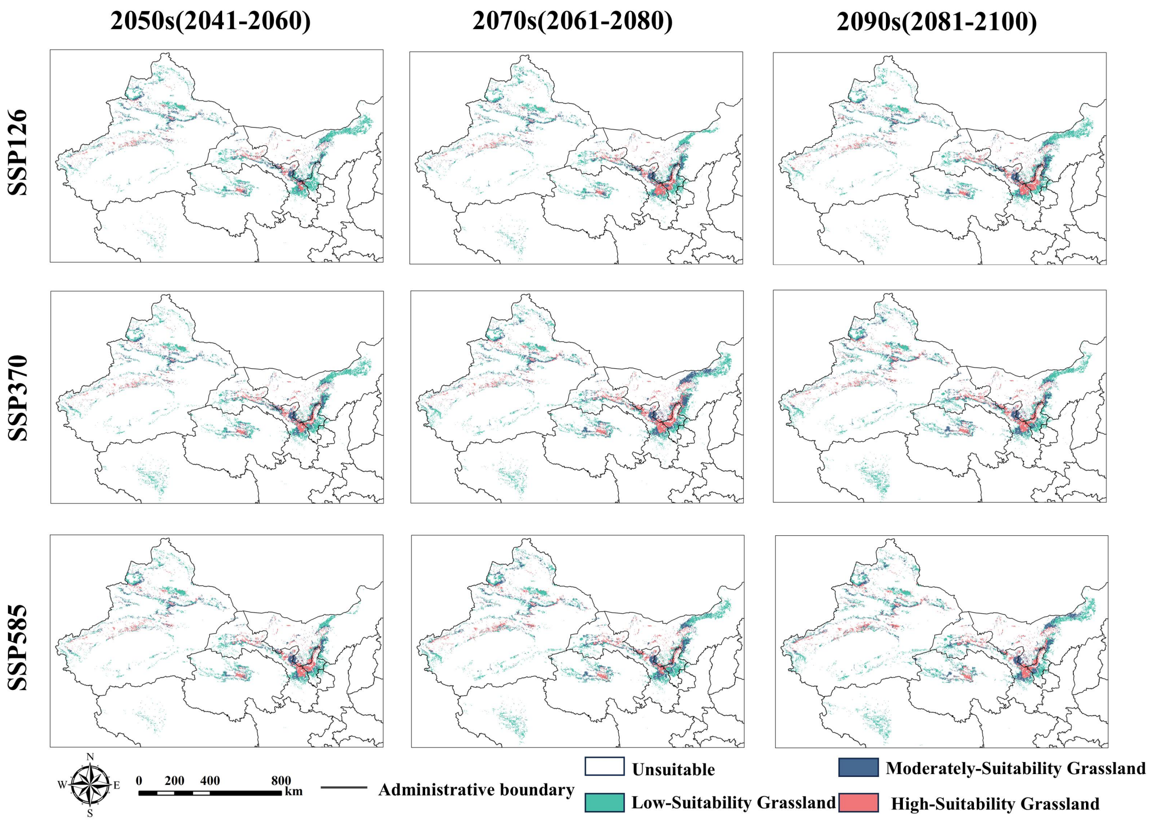

Fig S10. Distribution of Suitable Areas for *C. deserticola* in Grassland

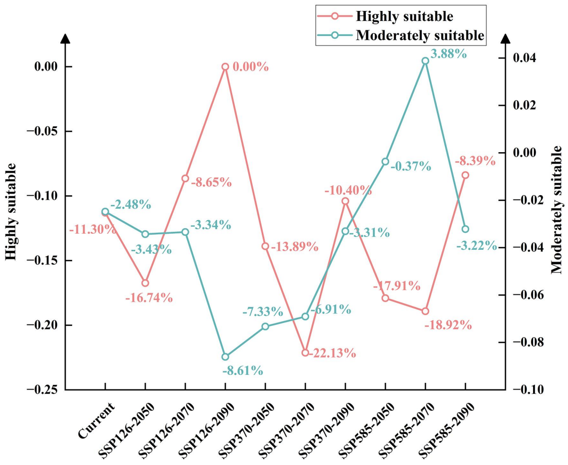

Fig S11. Area difference rate of medium and high suitability zones for C. deserticola under two scenarios
